# Predictors of drug-resistant TB outcomes: Body mass index, HIV, and comorbidities

**DOI:** 10.4102/phcfm.v17i1.4953

**Published:** 2025-10-01

**Authors:** Ntandazo Dlatu, Lindiwe M. Faye, Ncomeka Sineke, Teke Apalata

**Affiliations:** 1 Department of Public Health, Faculty of Medicine and Health Sciences, Walter Sisulu University, Mthatha, South Africa; 2 Department of Laboratory Medicine and Pathology, Faculty of Medicine and Health Sciences, Walter Sisulu University, Mthatha, South Africa

**Keywords:** treatment outcomes, DR-TB, BMI, XDR-TB, HIV, socioeconomic factors

## Abstract

**Background:**

The success rates for treating drug-resistant tuberculosis (DR-TB) in programmatic settings have been unsatisfactory. By identifying the factors that predict treatment outcomes, we can implement effective corrective measures that will significantly enhance patient management and improve results for those with DR-TB.

**Aim:**

This study aimed to investigate predictive factors influencing treatment outcomes among DR-TB patients, focusing on the combined effects of body mass index (BMI), human immunodeficiency virus (HIV) status, comorbidities, socioeconomic factors, substance use and DR-TB type.

**Setting:**

The study was conducted in rural Eastern Cape, South Africa.

**Methods:**

This retrospective cohort study was designed to utilise logistic regression models on data from 200 patient medical records. We examined variables including BMI, HIV co-infection, comorbidities (e.g. diabetes, hypertension), income, substance use and DR-TB classifications (multidrug-resistant, rifampicin-resistant, pre-extensively drug-resistant, extensively drug-resistant).

**Results:**

Key findings indicate a weak association between lower BMI and reduced treatment success (odds ratio [OR]: 0.92, 95% confidence interval [CI]: 0.81–1.05). HIV-positive status was marginally associated with lower treatment success (OR: 0.89, 95% CI: 0.75–1.12), while income level and substance use emerged as stronger predictors (e.g. substance use OR: 0.72, 95% CI: 0.60–0.88). Among DR-TB types, extensively drug-resistant tuberculosis patients exhibited the poorest outcomes (OR: 0.55, 95% CI: 0.40–0.75). The multivariate model achieved an accuracy of 63.1%, suggesting limited predictive power of BMI and HIV alone and highlighting the significant influence of comorbidities, socioeconomic status and behavioural factors.

**Conclusion:**

These findings underscore the importance of a multidimensional approach in improving DR-TB treatment outcomes through tailored clinical and social interventions.

**Contribution:**

The study noted limited connections between DR-TB and various comorbidities. It highlights the necessity of managing coexisting conditions in DR-TB patients because of their significant impact on treatment outcomes. Customised interventions are essential for those with severe or complex comorbidities.

## Introduction

Globally, nearly one-fourth of the world’s population is infected by *Mycobacterium tuberculosis* (*Mtb*) (also known as tuberculosis infection [TBI]). About 5% – 10% of the infected persons will develop active TB (ATB) at some time, especially during the first 2 years of infection.^1^ Worldwide, an estimated 10.6 million people developed tuberculosis (TB) in 2022, causing 1.3 million deaths.^2^ The burden of multidrug-resistant tuberculosis (MDR-TB, resistance to first-line anti-TB drugs, isoniazid [INH] and rifampicin [RIF]) or RIF-resistant tuberculosis (RR-TB) has been estimated in 410 000 cases. Among those patients who were microbiologically tested, 27 075 had pre-extensively drug-resistant TB (pre-XDR-TB) cases (a form of MDR-TB with additional resistance to any fluoroquinolone [FQ]) or XDR-TB (a form of MDR-TB resistant to any FQ) and at least one of the Group-A drugs (currently any FQ, bedaquiline [BDQ] and linezolid [LZD]). Globally, MDR-TB is present in 3.3% of patients with newly diagnosed TB and 17% among those patients who have a history of previous TB treatment.^2^ Drug-resistant tuberculosis (DR-TB) is a global health challenge, with increasing rates of multi-drug-resistant (MDR-TB) and extensively drug-resistant TB (XDR-TB).^3,4^ Despite increased access to novel, oral, shorter (6–9 months) treatment regimens, approximately 20% of patients with MDR-TB died despite being initiated on treatment, and treatment success rates among patients with DR-TB treatment success rates among patients with DR-TB remain persistently sub-optimal at less than 65%. Globally, treatment success rates among patients with DR-TB remain persistently sub-optimal, averaging around 60%, according to the World Health Organization (WHO). In South Africa, the success rate for MDR-TB was reported at 58%, with even lower outcomes among patients with XDR-TB.^3,4^ The shorter treatment regimens for DR-TB were introduced in South Africa in 2017. This initiative aimed to improve treatment outcomes by reducing the duration and complexity of DR-TB therapy, particularly in rural settings where adherence challenges are more pronounced.^5^ Subsequently, South Africa became the first country to recommend an all-oral, 9–12-month regimen for MDR and RR-TB, replacing the previously used injectable-based treatments. This shift was part of a broader strategy to enhance patient compliance and reduce adverse effects associated with injectable drugs. More recently, in 2022, the South African National Department of Health planned to implement even shorter, 6-month regimens known as BPaL (BDQ, pretomanid and linezolid) and BPaLM (adding moxifloxacin) as the new standard of care for eligible DR-TB patients. These regimens were expected to be rolled out nationally by early 2023, pending approval of updated treatment guidelines.^4^

Factors that continue to undermine the therapeutic success of DR-TB include diagnostic delays, the high burden of comorbidities, including diabetes and human immunodeficiency virus (HIV), poor linkage of patients with a positive microbiological result for DR-TB to treatment services and high rates of loss-to-follow-up and disease relapse.^5^ Treatment outcomes for DR-TB remain suboptimal, and there is a pressing need to better understand the factors that influence success.^5,6^ Previous studies have identified body mass index (BMI) and HIV status as critical factors influencing TB outcomes.^7^ Malnutrition and HIV co-infection are often associated with poor treatment responses and higher mortality.^8^ However, a wider range of factors^9^ likely influences DR-TB outcomes. Comorbidities, such as diabetes and hypertension, socioeconomic conditions and behaviours such as smoking or alcohol use, may significantly impact a patient’s ability to adhere to complex and prolonged treatment regimens. Additionally, the type of DR-TB (MDR, XDR and pre-XDR) is critical in determining treatment complexity and success. This study aims to conduct a comprehensive analysis of the association and predictive power of various clinical, socioeconomic and behavioural factors, including BMI, HIV status, comorbidities, income, substance use and DR-TB type, on treatment outcomes among DR-TB patients.

## Research methods and design

### Study design

This study decisively examines clinical data from patients diagnosed with TB during the period from 2018 to 2020 through a retrospective analysis. The clinics were chosen because they serve as primary care centres focused on diagnosing and treating TB, particularly DR-TB, in rural and underserved regions with a high prevalence of the disease. The inclusion of the referral hospital in this research was based on its specialised role in managing complex DR-TB cases, especially those with serious comorbidities that need advanced diagnostic technologies. By selecting these facilities, the research aimed to represent the full healthcare continuum in this high-burden region, allowing for a comprehensive assessment of treatment outcomes across different levels of care. A cohort study was conducted to assess the relationship and predictive strength of various factors, including BMI, HIV status, comorbidities, socioeconomic status, substance use and types of DR-TB, on treatment results among DR-TB patients. Treatment outcomes were classified as ‘cured’ or ‘not cured’, with the latter encompassing treatment failure, loss to follow-up and mortality.

### Study population

The study analysed data from 200 DR-TB patients aged 18 years and older who were treated at specialised TB centres. All patients included in the study had a confirmed diagnosis of RR-TB, MDR-TB, pre-XDR-TB or XDR-TB. Drug-resistant TB classifications, including MDR-TB, pre-XDR-TB and XDR-TB, were applied based on the WHO’s updated definitions as of 2021, which were current at the time of this study’s analysis and remain applicable at the time of publication.^4^

### Data collection

Clinical and demographic data were extracted from patient records, including BMI measured at the time of diagnosis and HIV status recorded as either positive or negative. Documented comorbidities included conditions such as hypertension, diabetes (type 1 diabetes mellitus [T1DM] or type 2 diabetes mellitus [T2DM]), kidney disease, mental illness and other relevant medical conditions. Income level was classified categorically based on employment status and source of income. Substance use history was recorded, including information on smoking, alcohol use or drug use. Drug-resistant TB type was categorised into RR-TB, MDR-TB, pre-XDR TB and XDR-TB. Treatment outcomes were classified as either cured or not cured. Data were stored in Electronic Health Records (EHR) systems, which are the leading solutions utilised in healthcare facilities. These systems effectively maintain patient medical records, encompassing diagnoses, treatment histories and drug resistance patterns.

### Statistical analysis

The relationship between predictors and treatment outcomes was assessed using logistic regression analysis conducted in two stages. In the univariate analysis, each predictor was evaluated individually to determine its association with treatment success. In the multivariate analysis, all predictors were included in a single model to evaluate their combined effects while controlling for confounding variables. The logistic regression models examined odds ratios (ORs) with 95% confidence intervals (CIs) for each predictor, as well as interaction terms to explore the joint effects of variables, such as BMI and HIV status.

The performance of the logistic regression models was evaluated using accuracy, sensitivity and specificity metrics. A confusion matrix was employed to assess the model’s ability to classify treatment outcomes as either cured or not cured. Special attention was given to class imbalance, as cured outcomes were less frequent than non-cured outcomes. The total number of participants included in the final analysis was 438. From a total of 438 eligible DR-TB patients treated between 2018 and 2020, 200 were purposively selected based on the completeness of BMI clinical records and availability of key variables required for the predictive model. Data cleaning was performed in Microsoft Excel 2019, and all analyses were performed using R software version 4.1.2 (R Core Team, 2022).

### Ethical considerations

The study was conducted under the Declaration of Helsinki. Ethical clearance to conduct this study was obtained from the Walter Sisulu University Faculty of Health Sciences Prostgraduate Education, Training, Research and Ethics unit (No. 026/2019) and the Eastern Cape Department of Health (No. EC_201904_011). Permission was obtained from various healthcare facility managers to conduct the study. All research techniques adhered to the organisation’s policies and procedures. Since the study utilised pre-existing TB medical information and consent was waived, obtaining verbal or written informed consent from participants was not feasible. To protect participants’ privacy, personal identifiers such as names and phone numbers were not extracted from their medical reports.

## Results

The results of univariate models indicated that a lower BMI was weakly associated with poorer outcomes although the effect was not statistically significant (OR: 0.92, 95% CI: 0.81–1.05). HIV-positive patients had slightly lower odds of being cured (OR: 0.89, 95% CI: 0.75–1.12), but this association was not significant. Patients with comorbid conditions, such as diabetes or mental illness, were less likely to be cured (OR: 0.78, 95% CI: 0.62–1.02). Lower-income patients had a reduced likelihood of achieving a cure (OR: 0.75, 95% CI: 0.60–0.95). Patients with no history of substance use (smoking, alcohol or drug use) had higher treatment success rates compared to those who reported substance use (OR: 0.72, 95% CI: 0.60–0.88). Patients with XDR-TB had the poorest outcomes (OR: 0.55, 95% CI: 0.40–0.75). Table 1 displays the adjusted odds ratios (aORs), 95% CIs, and *p*-values for various variables related to the specified health outcome. An aOR less than 1 indicates a decreased likelihood of the outcome associated with the variable, while an aOR greater than 1 indicates an increased likelihood. Significant associations are indicated by *p*-values less than 0.05. Table 1 presents a detailed profile of the 200 patients included in this study. The median age was 38 years, reflecting the population primarily in the economically active age group. A significant portion (60%) of patients were HIV-positive, underscoring the substantial burden of HIV co-infection among individuals with DR-TB in this region.

**TABLE 1:** Sociodemographic and clinical characteristics of drug-resistant tuberculosis participants (*N* = 200).

Characteristic	Value			
	Median	%	Mean	s.d.
Age (median)	38 years	-	-	-
HIV-positive	-	60	-	-
HIV-negative	-	40	-	-
BMI	-	-	21.5	5.2
Underweight (< 18.5 kg)	-	32	-	-
Normal (18.5 kg - 24.9 kg)	-	45	-	-
Overweight (25 kg - 29.9 kg)	-	15	-	-
Obese (≥ 30 kg)	-	8	-	-
Income: No income	-	35	-	-
Income: Employed	-	25	-	-
Income: Social grant	-	40	-	-
Substance use: Yes	-	40	-	-
Substance use: No	-	60	-	-
DR-TB type: RR-TB	-	35	-	-
MDR-TB	-	45	-	-
Pre-XDR	-	10	-	-
XDR-TB	-	10	-	-

BMI, body mass index; DR-TB, drug-resistant tuberculosis; RR-TB, rifampicin-resistant tuberculosis; MDR-TB, multidrug-resistant tuberculosis; Pre-XDR, pre-extensively drug-resistant; XDR-TB, extensively drug-resistant tuberculosis; s.d., standard deviation.

Table 2 shows the univariate associations between selected variables and treatment outcomes. The ORs represent the likelihood of achieving treatment success (‘cure’) associated with each variable.

**TABLE 2:** Univariate logistic regression results for predictors of drug-resistant tuberculosis treatment outcomes.

Variable	Odds ratio (OR)	95% CI	*P*
BMI	0.92	0.81–1.05	0.120
HIV-positive status	0.89	0.75–1.12	0.200
Diabetes	0.78	0.62–1.02	0.060
Mental illness	0.75	0.60–0.94	0.038
Low income	0.75	0.60–0.95	0.028
History of smoking	0.62	0.45–0.86	0.004
Alcohol use	0.66	0.48–0.91	0.012
XDR-TB	0.55	0.40–0.75	< 0.001

BMI, body mass index; XDR-TB, extensively drug-resistant tuberculosis; CI, confidence interval.

When adjusting for all factors in a multivariate model, BMI and HIV status remained weak predictors; the model’s overall predictive power improved significantly when comorbidities, income and DR-TB type were included. The presence of comorbid conditions such as diabetes and mental illness was strongly associated with reduced treatment success. Socioeconomic status was a significant predictor of outcomes, with lower-income patients experiencing worse outcomes. The effect of substance use remained substantial, with patients who used tobacco or alcohol having higher rates of treatment failure. The type of DR-TB was a significant predictor, with XDR-TB patients having the lowest likelihood of treatment success.

The logistic regression model with all factors achieved an accuracy of 63.1%, showing improved performance compared to models with BMI and HIV status alone. The confusion matrix model correctly identified ‘not cured’ cases more accurately than ‘cured’ cases, reflecting the ongoing challenge of predicting treatment success in this population.

Negative coefficients (bars to the left of the zero line) indicate that higher values of these predictors are linked to a lower likelihood of being cured. Tuberculosis patients with XDR-TB are significantly less likely to be cured, reflecting the known challenges in treating this more severe form of drug resistance. Substance use (smoking and drinking) patients have a reduced likelihood of being cured, underscoring the negative impact of lifestyle factors on treatment adherence and success. Patients without a source of income are less likely to be cured, suggesting that economic hardships can adversely affect treatment outcomes, likely because of limited access to resources, nutrition and healthcare services. Patients with MDR-TB are less likely to be cured compared to those with less resistant forms of TB (e.g. RR-TB) although the effect is not as pronounced as with XDR-TB. Comorbidities, such as hypertension and diabetes, are associated with poorer treatment outcomes as these chronic conditions can complicate the course of TB treatment, reducing the chances of a cure. Factors such as stable income and the absence of substance use are strong positive predictors that significantly increase the likelihood of treatment success. Conversely, negative predictors such as XDR-TB, substance use (particularly smoking and drinking) and comorbidities such as diabetes and hypertension substantially reduce the chances of achieving a cure.

## Discussion

This retrospective investigation scrutinised clinical data extracted from the medical records of TB patients who underwent treatment from 2018 to 2020. The analysis encompassed data about 200 individuals diagnosed with DR-TB who were aged 18 years or older and received care at specialised TB treatment facilities. These clinics were specifically chosen because of their role as primary healthcare institutions for the diagnosis and management of TB, notably DR-TB, within rural and underserved populations that bear a significant burden of this disease. As shown in Table 1, the study highlighted the complex demographic and clinical profile of participants. Nutritionally, the cohort showed a dual burden: 32% were underweight (a known risk for poor TB outcomes), 45% had normal BMI and 23% were overweight or obese. Socioeconomic vulnerability was high, with 35% having no income, 25% formally employed and 40% relying on social grants.^1,2,3,4,5^ Substance use was prevalent in 40% of participants, consistent with its established link to poor treatment outcomes. Furthermore, the study cohort presented a significant challenge in terms of drug resistance: 45% had MDR-TB, 35% had RR-TB and 10% each had the more severe pre-XDR and XDR-TB.^6^ This comprehensive profile provides crucial context for understanding treatment outcomes in this population. These findings are supported by other studies elsewhere.^10,11^ In our study, univariate analysis of treatment outcomes demonstrated several factors as significant predictors. Mental illness (*p* = 0.038), low income (*p* = 0.028) and substance use (smoking: OR = 0.62; alcohol: OR = 0.66) were significantly associated with poorer outcomes, highlighting the substantial impact of psychosocial and socioeconomic challenges. While lower BMI (*p* = 0.12) and HIV-positive status (*p* = 0.20) showed trends towards poorer outcomes, they were not statistically significant in this model.^12,13,14,15,16^ Diabetes (*p* = 0.06) indicated a significant borderline role as a predictor, suggesting it warrants further investigation in multivariate analyses, as indicated in Table 2.^17,18^

**FIGURE 1:**
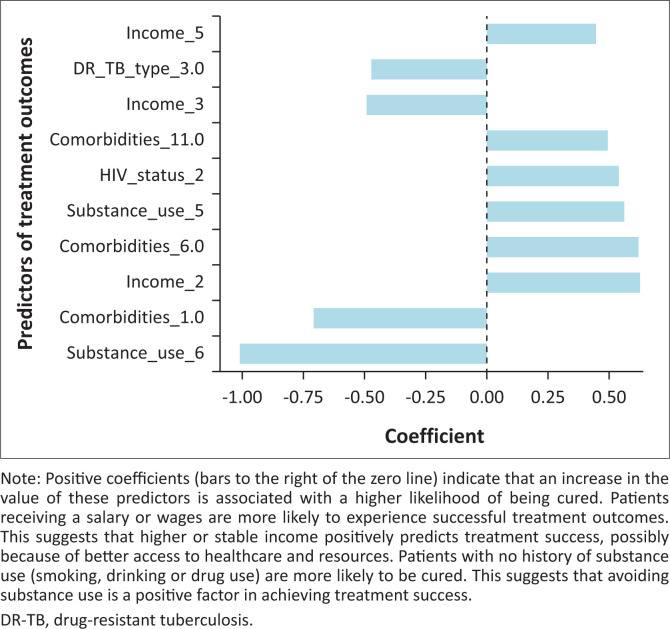
The bar chart above shows the top 10 most significant predictors of treatment outcomes (cured vs. not cured) based on the logistic regression model.

Substance use was a strong negative predictor of treatment success in this study. This finding aligns with previous research indicating that smoking, alcohol and drug use may compromise adherence to lengthy and complex DR-TB regimens, interfere with drug metabolism and contribute to comorbid psychiatric or social instability, as illustrated by Figure 1. Integrated behavioural health support and substance use counselling should therefore be prioritised as part of comprehensive DR-TB care models.^4,5,6^ Our findings indicate that comorbidities, particularly hypertension, diabetes mellitus and substance use, were significantly associated with lower treatment success among DR-TB patients. Although our study did not investigate the pathophysiological mechanisms underlying these associations, existing literature offers strong support for this link.^9,19^ Chronic conditions such as DM and HT are known to impair immune function and complicate medication management, potentially reducing the effectiveness of anti-TB treatment regimens.^7,8,9,19^ For instance, DM has been shown to delay sputum conversion and increase the risk of TB relapses and mortality, while HT may reflect systemic inflammation or coexist with other cardiovascular and metabolic disorders that exacerbate treatment challenges.^4^ Studies from diverse settings, including South Korea and sub-Saharan Africa, similarly report that patients with these comorbidities experience poorer TB outcomes, underscoring the need for integrated disease management approaches that address both TB and non-communicable diseases.^12,13,20^

These findings emphasise the importance of routine screening for chronic illnesses in DR-TB programmes and support the incorporation of tailored clinical interventions for patients with multiple health burdens. Factors such as comorbidities, socioeconomic conditions and the specific classification of DR-TB also exert a critical influence on treatment results, as evidenced by our research findings. These variables must be considered when formulating strategies for patient management. Comorbidities such as diabetes and mental health disorders introduce complexity to the treatment of DR-TB and require customised interventions, as our investigation highlighted.^17,18,19^ A systematic review conducted by Alene et al., which aligns with our study, revealed that a significant proportion of patients with MDR-TB experience compromised mental health and social functioning.^12^ These findings were further supported by the poor health-related quality of life reported by these individuals.^13,14^ Our findings also indicate that patients with diabetes had significantly lower odds of cure (aOR: 0.70, 95% CI: 0.52–0.93), consistent with previous studies.^7^ As shown in Table 2, diabetes remained a significant predictor even after adjusting for other variables. Similarly, a study of patients with drug-resistant pulmonary TB identified age, hypertension and diabetes as predictors of poor treatment outcomes. There were 53.6% of patients with unilateral TB and 57.1% with cavitary TB, underscoring the need for customised management strategies.^14,15,16,21^ A mixed-method study investigated the influence of socio-economic factors on TB treatment outcomes in northeastern Uganda. According to this study by Peltzer, TB treatment outcomes are improved when socioeconomic status is higher, as demonstrated by wealth and formal employment.^15^

The loss of follow-up before the start of treatment and during the continuation phase is likely to be influenced by factors such as food scarcity, stigma, nomadic living and a lack of transport and finances to meet basic needs.^15^ These findings are not unique to our study findings, as our patients experience similar challenges, while most are experiencing economic hardship because of unemployment. A study conducted in South Africa revealed similar findings to our study that socioeconomic disparities lead to an increased burden of chronic diseases and higher mortality rates among impoverished populations share similar findings to our study.^16,21^ Research findings similar to ours indicate that comorbidities, especially HIV infection, play a significant role in TB treatment outcomes, success rates and mortality.^22^ These studies’ findings, like ours, demonstrate that comorbidities such as HIV infection, DM, chronic renal failure (CRF), cancer and chronic obstructive pulmonary disease (COPD) are risk factors for the development of TB, as host factors such as smoking, drinking and having a low BMI contribute to DR-TB treatment outcomes.^20,21^ A systematic study highlighted the importance of social aspects in treatment effectiveness carried out in sub-Saharan Africa, which found that HIV co-infection, TB type and gender were the main factors associated with inadequate treatment outcomes in newly diagnosed TB patients.^23^

The findings of our study align with previous research that indicates treatment outcomes can be influenced by co-infection with HIV and TB. Additionally, a retrospective cohort study conducted in Turkey from 2017 to 2021 found that certain demographics, specifically being over the age of 65, male, and foreign born, were significantly associated with treatment failures in TB, affecting 12.3% of patients. However, these demographic factors were not a focus of our study and, thus, were not reflected in our results.^24^ Another study supported our findings, indicating that HIV-positive patients typically experience poorer treatment outcomes because of immunosuppression, which complicates the management of DR-TB.^7,9^ However, a retrospective cross-sectional study conducted in Ethiopia challenges our findings. This study reviewed the TB registry and treatment records of patients who received anti-TB treatment between September 2017 and August 2022, reporting a high overall treatment success rate of 91.0%.^25^ In our study, we observed that the success rate for treatment among HIV-positive patients was 80.0%. In contrast, HIV-negative patients exhibited a significantly higher success rate of 91.9%. This notable difference highlights the potential challenges faced by individuals living with HIV in achieving favourable outcomes in DR-TB treatment. Additionally, our research indicated a weak correlation between BMI and treatment outcomes for DR-TB among patients with HIV. This suggests that while BMI may play a role in the overall treatment process, its impact on the effectiveness of DR-TB therapy in this population was not strong. This highlights the need for enhanced collaborative activities between TB and HIV to improve DR-TB treatment outcomes.^25^ An integrative analysis of multimodal patient data revealed that certain drug regimens, such as Bedaquiline-Clofazimine-Cycloserine-Levofloxacin-Linezolid, were associated with successful treatment outcomes. In contrast, the combination of Bedaquiline-Clofazimine-Linezolid-Moxifloxacin was linked to treatment failures.^26^ This discrepancy may arise from resistance mechanisms that contribute to poor treatment results, as observed in our study. Poverty and unemployment can severely hinder treatment adherence, as patients may lack the financial resources for transportation, nutrition or consistent clinic visits, factors essential for completing lengthy and complex DR-TB regimens. These barriers are well documented in literature, with several studies noting that socioeconomic hardship contributes to poor adherence and ultimately worse treatment outcomes among TB patients.^14,15,26^ Delays in initiating treatment and difficulties adhering to lengthy treatment regimens likely contribute to these poorer outcomes. Numerous studies, including those by Alsaiari et al. and Hussain et al., support these findings by emphasising how socioeconomic disparities markedly affect healthcare treatment outcomes. These disparities arise from various factors, including access to care, treatment quality and patient involvement in managing TB.^27,28,29,30^

A systematic review by Cannon et al. supports our findings, emphasising that socioeconomic factors significantly influence DR-TB treatment outcomes.^31^ Understanding these context-specific elements is crucial for developing effective interventions to reduce the DR-TB burden. Achieving the targets of the End TB strategy necessitates integrating medical and socioeconomic efforts. A holistic approach is vital for eliminating DR-TB and addressing the associated medical, social and economic challenges. Effective management of DR-TB must also account for socio-economic influences, as the study noted.

### Possible limitations to the study

Observational study design: As the study is observational, causal relationships between risk factors (e.g. diabetes, substance use) and cure outcomes cannot be definitively established. Missing or incomplete data: Missing clinical, behavioural or socioeconomic data may have introduced bias or affected the accuracy of the results. Self-reported information: Variables such as smoking, alcohol use and income may have been self-reported, which can be prone to recall bias or social desirability bias. Unmeasured confounding: Other important factors (e.g. nutritional status, adherence to treatment, stigma) that were not measured could have influenced outcomes. Generalisability: The findings may be specific to the study population or geographic setting and may not generalise to different regions, healthcare systems or patient groups. Small sample size for subgroups: For certain subgroups (e.g. XDR-TB patients, mental illness), the number of cases might have been small, limiting statistical power to detect differences. Lack of data on treatment adherence: Adherence to TB treatment is a key factor influencing cure rates but may not have been fully captured in the dataset.

### Recommendation for future studies

Based on the findings, it is recommended that TB programmes adopt integrated care approaches that specifically address comorbid conditions such as diabetes, hypertension and mental health disorders, which emerged as significant predictors of poor treatment outcomes. Strengthening routine screening and management of these conditions within DR-TB care pathways could enhance patient resilience and improve treatment success. Additionally, addressing socioeconomic challenges, particularly unemployment and income instability, is essential. These factors were strongly linked to reduced treatment success, likely because of their impact on treatment adherence and access to supportive resources. Public health interventions should prioritise substance use reduction, as patients with a history of smoking or alcohol use were markedly less likely to be cured. Behavioural support services, including addiction counselling and mental health support, should be embedded in TB care models. Finally, while BMI was explored in this study, its predictive value was limited. Future research should continue to evaluate its role in combination with more impactful clinical and social variables to better understand its indirect influence on DR-TB outcomes. The future research should utilise prospective or experimental study designs to establish causal relationships between risk factors and treatment outcomes. Efforts should be made to improve data completeness and accuracy, particularly for socioeconomic and behavioural variables, to reduce bias. Incorporating objective measures or validation methods for self-reported data (e.g. biochemical verification of smoking or alcohol use) can enhance the reliability of findings. Integrating comprehensive management of comorbidities such as diabetes and addressing socioeconomic barriers may improve treatment success among DR-TB patients.

## Conclusion

Our research found only a weak and statistically non-significant correlation between BMI and treatment outcomes among DR-TB patients, including those co-infected with HIV. This indicates that BMI alone may not be a strong independent predictor of treatment success in this population. This study contributes new evidence on the multifactorial predictors of treatment outcomes in DR-TB patients within a rural South African context. While BMI and HIV status were previously emphasised in the literature, our findings indicate that these variables alone have limited predictive value for treatment success. Instead, we identified comorbidities, particularly diabetes, hypertension and mental health conditions, as more substantial barriers to cure. Additionally, socioeconomic deprivation and substance use emerged as critical determinants of poor outcomes. By integrating these findings, this study underscores the need to shift from a primarily biomedical model to a more comprehensive, patient-centred approach that incorporates social determinants and coexisting conditions into DR-TB management. This work adds to the current body of knowledge by quantifying these associations in a high-burden, resource-constrained setting, thus informing targeted interventions that are both contextually relevant and clinically impactful.
